# A novel method for blood vessel detection from retinal images

**DOI:** 10.1186/1475-925X-9-14

**Published:** 2010-02-28

**Authors:** Lili Xu, Shuqian Luo

**Affiliations:** 1 School of Biomedical Engineering, Capital Medical University, Beijing, China

## Abstract

**Background:**

The morphological changes of the retinal blood vessels in retinal images are important indicators for diseases like diabetes, hypertension and glaucoma. Thus the accurate segmentation of blood vessel is of diagnostic value.

**Methods:**

In this paper, we present a novel method to segment retinal blood vessels to overcome the variations in contrast of large and thin vessels. This method uses adaptive local thresholding to produce a binary image then extract large connected components as large vessels. The residual fragments in the binary image including some thin vessel segments (or pixels), are classified by Support Vector Machine (SVM). The tracking growth is applied to the thin vessel segments to form the whole vascular network.

**Results:**

The proposed algorithm is tested on DRIVE database, and the average sensitivity is over 77% while the average accuracy reaches 93.2%.

**Conclusions:**

In this paper, we distinguish large vessels by adaptive local thresholding for their good contrast. Then identify some thin vessel segments with bad contrast by SVM, which can be lengthened by tracking. This proposed method can avoid heavy computation and manual intervention.

## Background

The retina is the only location where blood vessels can be directly captured non-invasively in vivo. Over the past decade, the retinal image analysis has been widely used in medical community for diagnosing and monitoring the progression of diseases [1,2]. And retinal blood vessels are important structures in retinal images. The information obtained from the examination of retinal blood vessels offers many useful parameters for the diagnosis or evaluation of ocular or systemic diseases. For example, the retinal blood vessel has shown some morphological changes such as diameter, length, branching angles or tortuosity for vascular or nonvascular pathology, such as hypertension, diabetes, cardiovascular diseases [3]. Blood vessels are also used as landmarks for registration of retinal images of a same patient gathered from different sources. Sometimes, retinal blood vessel must be excluded for easy detection of pathological lesions like exudates or microaneurysms. In all cases, proper segmentation of retinal blood vessel is crucial.

Actually, automatic detection of the blood vessels in retinal images is a challenging task. The contrast of retinal image diminishes as distance of a pixel from the center of the retinal image. And the presence of noise, the variability of vessel width, the presence of some pathological lesions, all make the task more difficult.

There are three basic approaches for automated segmentation of blood vessel: thresholding method, tracking method and machine trained classifiers. In the first method, many of different operators are used to enhance the contrast between vessel and background, such as Sobel operators, Laplacian operators, Gaussian filters which model the gray cross-section of a blood vessel [4]. Then the gray threshold is selected to determine the vessel. And this gray threshold is crucial, because small threshold induces more noises and great threshold causes loss of some fine vessels, so adaptive or local threshold is used to different sections of an image.

Vessel tracking is another technique for vessel segmentation, whereby vessel centre locations are automatically sought along the vessel longitudinal axis from a starting point to the ending point [5]. This method may be confused by vessel crossings and bifurcations.

Many kinds of classifiers, such as Bayesian classifier, neural networks, support vector machine, have been employed for improved discrimination between vessel and non vessel. Feature extraction and parameters selection of a classier are critical. All pixels in images are classified into vessel or non-vessel through the classifier [6,7].

In fact, a single generally acknowledged vessel segmentation algorithm does not exist due to the unique properties of each acquisition technique. Every segmentation method has difficulties when applied alone, a combination of them is presented to detect retinal blood vessel in this paper. This article is organized as follows. Section 2 describes the method of segmentation of blood vessel. Section 3 shows the results. The discussions and conclusions are presented in Section 4.

## Methods

Due to the acquisition process, retinal images often have a variational gray level contrast. In general, large vessels display good contrast while the thin ones show bad contrast. Thereby pixels attached to large and thin vessels show the different gray level and geometrical correlation with the nearby pixels. So we extract large and thin vessels separately. The proposed method is made up of four fundamental parts, (1) preprocessing, which involves background normalization, image binarization and large vessel extraction, (2) feature extraction of fragments, which are the residual parts of binary retinal image with large vessels excluded, (3) classification of fragments, support vector machine is used to distinguish thin vessel segments from all the fragments, (4) thin vessel growth, based on tracking method.

### Image preprocessing and large vessels extraction

**Figure 1:**
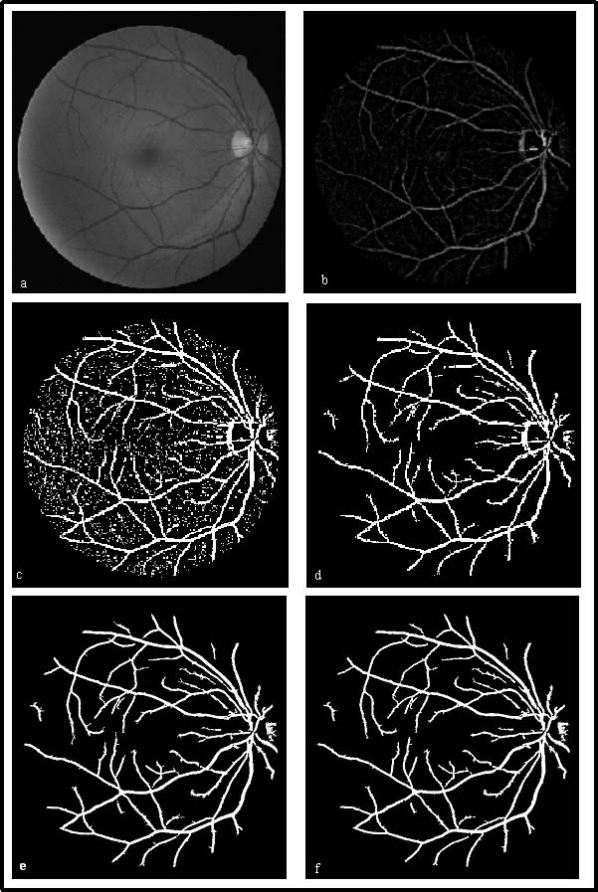
**Image preprocessing and large vessel extraction**. (a) Original gray image from green channel. (b) Normalized retinal image. (c) Binary retinal image. (d) Large vessels candidates. (e) Large vessels candidates with optic disk removed. (f) Obtained large vessels.

In the RGB images, the green channel exhibits the best contrast between the vessels and background while the red and blue ones tend to be more noise. So we work on the gray image from green channel and the retinal blood vessels appear darker in the gray image, shown in Fig.1(a). Normalization is performed to remove the gray-level deformation by subtracting an approximate background from the original gray image [8]. The approximate background is estimated using a 25 × 25 median filter on the original retinal image. Thereby blood vessels are brighter than the background after normalization, as Fig.1(b).

The adaptive local thresholds are implemented to the normalized image, and the binary retinal image is shown in Fig.1 (c). It is obvious that the large connected components are the large vessels. So the large connected components, whose area are greater than *T *(*T *is the minimum grain size, and equals 100 in this paper), are reserved as the large vessels candidates, shown in Fig.1(d).

The edges of the optic disk are usually mistaken for large vessels. According to the spatial gray properties of the optic disk and large blood vessels, automatic erasing the edges of the optic disk are implemented subsequently. The optic disk is the origin of blood vessels and the brightest region in retinal image. Large vessels are dark objects with two-sides boundary, relative to the background. In the gradient images convoluted with Sobel operators along horizontal and vertical directions, a large vessel always corresponds to a pair of local gradient maximum and minimum on both sides along a profile. And the edge of the optic disk corresponds to a single local maximum or minimum. We locate the optic disk, reserve the pixels between a pair of local gradient maximum and minimum around the optic disk along horizontal or vertical direction and remove all the small objects nearby. Fig.1(e) shows the binary retinal image where the edges of the optic disk are erased automatically.

As mentioned above, a large vessel corresponds to a pair of local gradient maximum and minimum on both sides. So we exclude the pixels outside the pairs of extrema and fill in the inner parts. By doing this, we obtain the large blood vessels, shown in Fig.1(f).

The residual fragments of the binary image are not all noises, shown in Fig.3(a). They should be classified by support vector machine next.

### Feature extraction

The wavelet and curvelet transform are all multiscale transforms. They are now recognized as useful feature extraction methods to represent image features at different scales. And the wavelet and curvelet transforms exhibit impressive performance in detecting point and line features, respectively. Nevertheless, the wavelet and curvelet modulus are bigger along the image edges.

A wavelet transform can be defined as Eq.1, where *Ψ*_*a, b *_is the wavelet with scale *a*, location *b *by dilations and translations from mother wavelet *Ψ *centered on the origin. Similarly, a curvelet transform can be defined as Eq.2. Here *Ψ*_*a, b, θ *_is a curvelet with scale *a*, location *b *and direction *θ*, which can be regarded as a wavelet *Ψ*_*a, b *_stretched preferentially in direction *θ *[9].(1)

However, two dimensional discrete wavelet transform can be implemented by consecutive low-pass and high-pass filters through convolution along horizontal and vertical directions. After one-level decomposition on an original image, an approximate sub-image and three detail sub-images are obtained. Those detail sub-images are at horizontal, vertical and diagonal orientation, respectively. Down- sampling is discarded in wavelet transform for sub-images with the same size of the original image to facilitate feature extraction on each pixel in residual image. Then we calculate wavelet modulus *M*_*W *_by Eq.3, where *W*_*v *_and *W*_*h *_are the horizontal and vertical sub-image with Haar wavelet transform, respectively. A fast curvelet transform can be implemented via 'wrapping', as this is the fastest curvelet transform algorithm currently available. The curvelet transform decomposes the image into curvelet subbands with different sizes. In this paper, the original gray image, decomposed using curvelet transform at scale 2 and angle 8, will produce one approximate (coarse) subband and eight detailed coefficient blocks. We select each detailed coefficient block to reconstruct image, denoted *C*_*i *_(*i *= 1, 2Λ 8), and calculate the modulus image *M*_*C *_by Eq.4.(3)

**Figure 2:**
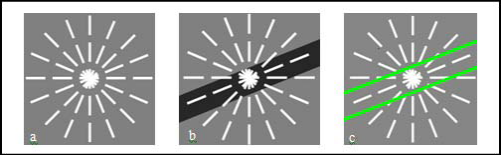
**Basic line detectors and identification blood vessel orientation**. (a) Eight basic line detectors. (b) Identification of blood vessel orientation in gray image. (c) Identification of blood vessel orientation in modulus image, where the brightened green lines stand for the modulus of vessel edges.

As concerns the thin vessels ignored in large vessel extraction, they can be regarded as the lines with width within 3 pixels. So some basic line detectors are used to identify the orientation of thin vessels, shown in Fig.2(a). We consider 8 line detectors of fixed length *l *passing through each residual pixel (*x*, *y*) at different 8 angles (22.5× of angular resolution) [7]. And the mean and standard deviation of gray level is evaluated along each line. As shown in Fig.2(b), the mean and standard deviation of gray level along the line aligned within the vessel is minimum for almost invariable gray level. And this direction is marked by *D*1(*x*, *y*). The line with largest mean and standard deviation is found and corresponding direction is marked by *D*2(*x*, *y*). Now, we denote the mean and standard deviation of gray level along *Di*(*x*, *y*), with *M*_*gi*_(*x*, *y*) and *SD*_*gi*_(*x*, *y*), where *i *= 1 or 2. According to the orientation of thin vessel, *M*_*g*1_(*x*, *y*) and *M*_*g*2_(*x*, *y*), *SD*_*g*1_(*x*, *y*) and *SD*_*g*2_(*x*, *y*) differ significantly if pixel (*x*, *y*) is attributed to a thin vessel. Otherwise, *M*_*g*1_(*x*, *y*) and *M*_*g*2_(*x*, *y*), *SD*_*g*1_(*x*, *y*) and *SD*_*g*2_(*x*, *y*) differ slightly if (*x*, *y*) is non-vessel pixel. The difference of gray mean and standard deviation along *D*1(*x*, *y*) and *D*2(*x*, *y*) are calculated by Eq. 5 and 6. And these differences of vessel pixel are greater than the corresponding differences of non-vessel pixel. Similarly, the means and standard deviations of modulus of wavelet and curvelet coefficients differ significantly along *D*1 and *D*2 directions for vessel pixel, while they differ slightly for non-vessel pixels, shown as Fig.2(c). In the same way, we denote the mean and standard deviation of wavelet and curvelet modulus along *D*1(*x*, *y*), with *M*_*W*1_(*x*, *y*), *SD*_*W*1_(*x*, *y*), *M*_*C*1_(*x*, *y*) and *SD*_*C*1_(*x*, *y*), where subscript *W *and *C *represent wavelet and curvelet modulus, respectively. And *D*_*m*_*W*_, *D*_*sd*_*W*_, *D*_*m*_*C*_, *D*_*sd*_*C *_stand for the differences of the means and standard deviations of wavelet or curvelet modulus along *D*1(*x*, *y*) and *D*2(*x*, *y*) directions, respectively.(5)

Then we construct a 12 dimensional feature vector *x *= [ *M*_*g*1_, *SD*_*g*1_, *D*_*m*_*g*_, *D*_*sd*_*g*_, *M*_*W*1_, *SD*_*W*1_, *D*_*m*_*W*_, *D*_*sd*_*W*_, *M*_*C*1_, *SD*_*C*1_, *D*_*m*_*C*_, *D*_*sd*_*C*_] for each residual pixel in the binary retinal image excluded large vessels. A normal transformation is applied to the feature vector *x *according to Eq.7.(7)

where *x*_*i_ori *_is the original *i*th feature (*i *= 1, 2Λ 12) of *x*, while *x*_*i*_max _and *x*_*i*_min _are the maximum and minimum of *i*th feature. And the *x*_*i *_ranges from -1 to 1 after normalization.

### Classification based on support vector machine

Binary SVM was first suggested by Vapnik and widely used in pattern recognition application. It's based on strong foundations from the broad area of statistical learning theory according to structural risk minimization. SVM offers several advantages such as well performance in higher dimensional spaces. The basic idea of binary SVM is to find the hyper-plane that best separates vectors from both classes in feature space while maximizing the distance from each class to the hyper-plane. There are both linear and nonlinear approaches to SVM. If the two classes are linearly separable, SVM computes the optimal separating hyper-plane with the maximum margin by solving the following the optimization problem: minimize ||*w*||^2^, subjected to the constraint *y*_i_(*w*^T^*x *+ *w*_0_) ≥ 1, where *x *is the feature vector. If the two classes are nonlinearly separable, the slack variables ξ_*i *_are introduced. SVM finds the optimal separating hyper-plane by minimizing the following equation subjected to *y*_i_(*w*^T^*x *+ *w*_0_) ≥ 1-ξ_*i*_:(8)

Where the constant *C *> 0 is user defined and determines the trade-off between the maximization of the margin and minimization of the classification error [7].

The input vector should be mapped to a high dimensional features space by an inner-product kernel, named 'kernel function'. The selection of 'kernel function' is important to SVM. We compare the performances of commonly used kernel functions, such as Gaussian radial basis function (rbf), linear kernel, polynomial kernel and exponential Gaussian radial basis function (erbf). They provide approximately the same performance in test sensitivity and 'rbf' outperformed the others in test specificity. So the 'rbf' is used in further tests, given by:(9)

where *x *is the feature vector, *y *is the corresponding classification result and σ is width of 'rbf'. Here *y *is 1 as pixel attached to a thin vessel and -1 to non-vessel pixel.

In practical application, error classifications are at different risks for vessel and non-vessel pixel. The thin vessel can be detected by tracking growth as long as any pixel situated in the thin vessel is identified correctly. However, once the non-vessel pixel identified into thin vessel segment, it induces more noises after tracking growth. As a solution to this problem, different *C *can be assigned to two class pixels, namely, small *C*1 for vessel pixels and large *C*2 for non-vessel pixels, given in Eq.10.(10)

### Growth of thin vessel

The thin vessel segments have been discriminated from noises after classification. And the thin vessels can be detected by tracking growth from these isolated thin vessel segments. In this paper, we track a thin vessel form a vessel segment by combining *D*1 direction and eigenvector of the Hessian matrix. The Hessian matrix of the gray image *f*(*x*,*y*) is defined as(11)

where subscripts *x *and *y *denote the convolution of original gray image with the second order Gaussian derivate along *x *or *y *direction. Then the eigenvalues of Hessian matrix and their corresponding eigenvectors can be calculated. The eigenvalues, *λ*_1 _and *λ*_2_, where we take *λ*_1 _≥ *λ*_2_, measure convexity and concavity in the corresponding eigendirections. For a retinal image, where vessels are darker than the background, the eigenvalues of the Hessian matrix are λ_1 _>> 0 and λ_2 _≈ 0 for vessel pixels [10].

The endpoints of the thin vessel segments grow along *D*1 direction recursively, until the estimated next thin vessel endpoints can't meet the conditions of λ_1 _>> 0 and λ_2_≈ 0.

## Results and Discussion

This method is evaluated on the publicly available DRIVE database [11]. The DRIVE database contains 40 color retinal images. These 40 images were divided into a training set and a test set. The binary images of manual segmentation and the masks of field of view (FOV) are available for all the images of the two sets. All the images were manually segmented. And those of the test set were segmented twice by two experienced experts. And the 1^st ^expert marked 12.7% of pixels as vessel, against 12.3% for 2^nd ^expert. The performance of the different segmentation method is usually evaluated on the test set using the 1^st ^manual segmentations as ground truth. The 2^nd ^expert reaches the accuracy of 0.9473, which is usually used as references.

We first select a training set to build the SVM classifier. The training set is made up 2000 pixels, extracted in proportional spacing from the 20 residual images excluded large vessels in the training set (100 pixels per image). There are 1000 thin vessel pixels while the others are due to the presence of noise in the images. Classification performance is then evaluated on all pixels of the 20 residual images of the training set. In order to ensure accurate classification for most noise pixels (about >95%), the SVM is used continually until the number of remained pixel is less than 1000. According to the training set, there are about 1500 thin vessel pixels and more than 10000 non-vessel pixels in each residual image. And less than about 400 thin vessel pixels remained after SVM, so the specificity (ratio of the number of true negatives and the number of all negative samples) can't reach 95% if the reserved pixels are more than 1000.

**Figure 3:**
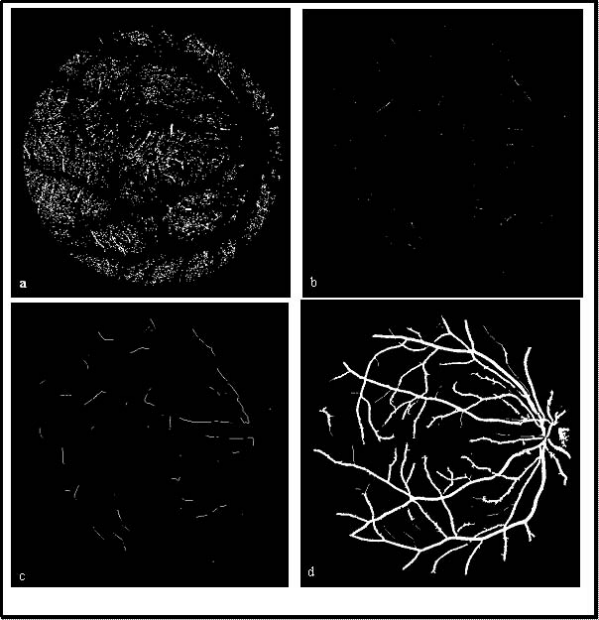
**The process of thin vessel detection**. (a) Residual fragments after large vessel removed. (b) Thin vessel segments after fragments classification. (c) Growth of thin vessel segments. (d) Whole vessels reserved.

Fig.3 shows the process of thin vessels extraction, (a) residual fragments after large vessels extraction of Fig.1(c), (b) the thin vessel segments identified by SVM, (c) thin vessel growth of (b), (d) the whole vessels reserved.

Table 1 presents the sensitivity (ratio of the number of true positives and the number of positive samples) and accuracy (ratio of the number of samples classified correctly and all samples) of each image in test set. To facilitate the comparison with other retinal vessel segmentation algorithms, we have selected the segmentation sensitivity and accuracy as performance measures, which are indicators of the number of properly classified pixels, respectively in the vessel pixels and all pixels in FOV. Table 2 compare our approach with some other vessel segmentation methods in the term of average accuracy and sensitivity, as obtained from the DRIVE database site [11]. Our method predominates over the other methods in higher sensitivity. However, relatively low accuracy is a shortage, which's due to the inflated width of large vessels in image preprocessing.

**Table 1:** Performance of segmentations blood vessel in test set

No.	1	2	3	4	5
sensitivity	0.8161	0.8046	0.7151	0.7108	0.7468
accuracy	0.9365	0.9404	0.9261	0.9410	0.9361

No.	6	7	8	9	10

sensitivity	0.7479	0.7274	0.7399	0.7646	0.7646
accuracy	0.9270	0.9329	0.9119	0.9340	0.9388

No.	11	12	13	14	15

sensitivity	0.7413	0.7975	0.7368	0.8190	0.8321
accuracy	0.9314	0.9305	0.9314	0.9300	0.9195

No.	16	17	18	19	20

sensitivity	0.7862	0.7602	0.8113	0.8416	0.8557
accuracy	0.9375	0.9349	0.9390	0.9434	0.9336

**Table 2:** Comparison with some different vessel segmentation methods

Method	Average accuracy (standard deviation)	Average sensitivity
2^nd ^expert	0.9473(0.0048)	0.7761
our method	0.9328(0.0075)	0.7760
Mendonca (green-channel)	0.9452(0.0062)	0.7344
Staal	0.9442(0.0065)	0.7194
Niemeijer	0.9417(0.0065)	0.6898

## Conclusions

In this paper, we deal with the retinal blood vessel that appears split into two parts, due to the contrast, large and thin vessels. The large vessels are detected by adaptive local thresholding in normalized images. Then the residual fragments including thin vessel segments are classified by SVM. This method avoids heavy computation that applied SVM to each pixel. And the thin vessel can be extracted by iterative linear extrapolation without manual given start points.

## Competing interests

The authors declare that they have no competing interests.

## Authors' contributions

LX worked on the algorithm design and implementation, and wrote the paper; SL contributed discussions and suggestions throughout this project, including the manuscript writing. All authors read and approved the final manuscript.
